# Circulating endothelial microparticles and *miR-92a* in acute myocardial infarction

**DOI:** 10.1042/BSR20170047

**Published:** 2017-03-27

**Authors:** Yuchen Zhang, Junjun Cheng, Fang Chen, Changyan Wu, Junmeng Zhang, Xuejun Ren, Yu Pan, Bin Nie, Quan Li, Yu Li

**Affiliations:** 1 Department of Cardiology, Beijing An Zhen Hospital of the Capital University of Medical Sciences, Beijing 100029, China, Anzhen Road, Chaoyang District; 2 Institute of Medicinal Biotechnology, Chinese Academy of Medical Sciences and Peking Union Medical College, Beijing 100050, China, Tian Tan Xili, Beijing

**Keywords:** acute myocardial infarction, microparticles, microRNAs

## Abstract

Microparticles (MPs) and miRNAs have been shown to play important roles in coronary artery disease (CAD) by monitoring endothelial dysfunction. The present study aims to investigate the diagnostic value of endothelial MPs (EMPs) and miRNAs (*miR-92a* or *miR-23a*) as biomarkers in distinguishing patients with acute myocardial infarction (AMI) from those with CAD. Plasma samples from 37 patients with AMI, 42 patients with stable CAD (SCAD), and 35 healthy adults were collected for investigation in the present study. The numbers of CD31+/CD42b− MPs, CD31+/CD42b+ MPs, and CD31−/CD42b− MPs were measured by flow cytometry and the levels of *miR-92a* and *miR-23a* were analyzed using reverse transcription-quantitative PCR. Moreover, cardiac troponin I (cTnI) expression was detected by ELISA to serve as a routine diagnostic parameter. The number of CD31+/CD42b− was higher in AMI group than those in SCAD and healthy groups. Besides, the expression of *miR-92a* was higher in AMI group compared with two other groups. Furthermore, evidence showed that there was a positive correlation between the levels of CD31+/CD42b− MPs and *miR-92a*. Finally, the receiver operating characteristic (ROC) curve revealed that the area value under the curve of CD31+/CD42b− MPs, *miR-92a* and cTnI was 0.893, 0.888, and 0.912 respectively. CD31+/CD42b− MPs and *miR-92a* might have great potential to provide diagnostic value for AMI and could probably regulate the endothelial dysfunction in AMI patients.

## Introduction

It is noteworthy that coronary heart disease, particularly acute myocardial infarction (AMI), is the leading cause of death and disability worldwide [1,2]. AMI is characteried by cardiac cell death resulted from exposure to prolonged ischemia after occlusion of a coronary artery. Rapid and correct diagnosis of AMI is believed to be a key role in therapy and prognosis for this disease [3]. At present, cardiac troponin I (cTnI) is the golden standard used by clinicians to diagnose patients. However, cTnI cannot distinguish between patients with stable coronary artery disease (SCAD) and patients at risk for AMI [4]. Therefore, it is essential to identify novel biomarkers with high sensitivity and specificity for early diagnosis of AMI.

Microparticles (MPs) are small membrane vesicles released from many different cell types in response to cellular activation or apoptosis. MPs shedding from endothelial cells are called endothelial MPs (EMPs) [5]. Some evidence suggest a higher expression of EMPs in patients with SCAD and AMI than that in healthy people [6]. However, little is known about the difference of EMPs expression between SCAD and AMI.

Moreover, in recent years, a large number of data suggest that microRNAs (miRNAs, miR) are important for the development of various disorders, including cardiovascular diseases [7,8]. The role of plasma miRNAs in AMI has been recently investigated [9]. Some evidence demonstrated that *miR-92a* and *miR-23a* could regulate endothelial dysfunction and atherosclerosis [10,11]. However, the expression difference of both *miR-23a* and *miR-92a* between SCAD and AMI is largely undetermined.

Therefore, the present study was designed to determine the diagnostic value of EMPs and *miR-92a* or *miR-23a* as biomarkers in distinguishing patients with AMI from those patients with coronary artery disease (CAD).

## Materials and methods

### Study subjects

We recruited 37 patients with AMI, 42 patients with SCAD, and 35 healthy adults in the present study from September 2015 to December 2015. Patients with SCAD were diagnosed based on presentation with exertional substernal chest pressure without recent acceleration in symptoms and evidence of a significant angiographic stenosis ≥70%, in the absence of myocardial necrosis. Patients with AMI were diagnosed according to the universal definition of myocardial infarction [12]. All subjects including patients and controls with a history and clinical features of infectious disease, auto-immune, malignant tumor, peripheral vascular disease, lung diseases, hepatic or hematologic disorders, or chronic renal failure requiring dialysis were excluded from the present study. The present study’s protocol was approved by the Ethics Committee (Beijing Anzhen Hospital of the Capital Univeristy of Medical Sciences) of and informed written consent was obtained from all these subjects. Clinical and laboratory data were collected from all subjects.

### cTnI determination

The preferred biomarker for each specific category of MI is cTnI, which has high myocardial tissue specificity as well as high clinical sensitivity. Detection of rise and fall of the measurements is essential to the diagnosis of AMI. An increased cTnI concentration is defined as a value exceeding the 99% of a normal reference population [13]. Blood samples were obtained after 1 h in the emergency department [14]. Plasma cTnI concentrations were determined by chemiluminescence immunoassays according to manufacturer’s protocol (Beckman Coulter, Fullerton, CA, U.S.A.).

### Sample preparation for MP analysis and miRNA measurement

Whole blood samples were collected into 15-ml sterile centrifuge tube containing citrate anticoagulant and centrifuged at 1500 ***g*** for 20 min. One milliliter of plasma was drawn carefully by a micropipette into a 1.5-ml sterile Eppendorf tube and centrifuged at 13000 ***g*** for 2 min to obtain the platelet-poor plasma (PFP). Then, samples were stored respectively at −200°C for a week and then at −80°C until MP analysis.

PFP samples were transferred into new RNase/DNase-free tubes and stored at −80⁰C until RNA extraction. It has been shown that miRNAs in frozen plasma remain stable for years and are reliable biomarkers of cardiac heart disease.

### MP detection through flow cytometry

The top 50 μl of PFP was then incubated with fluorochrome-labeled antibodies specific for CD31 (BD Biosciences) and CD42b (BD Biosciences) for 20 min in the dark at 4°C. Thereafter, the samples were diluted with 1 ml of PBS and then measured by flow cytometry. EMPs were identified as CD31+/CD42b− events within the MP size gate, platelet MPs (PMPs) as CD31+/CD42b+, and MPs derived from other cells as CD31−/CD42b−.

### miRNA analysis through qRT-PCR

Quantitative real-time PCRs (qRT-PCR)s were performed using the high-throughput BioMark Real-Time PCR system (Fluidigm, South San Francisco, CA, U.S.A.) according to the manufacturer’s protocol to determine the quality and abundance of miRNA. miRNA was quantified using NanoDrop spectrophotometer, and 10 ng of the total RNA was reversely transcribed using a TaqMan miRNA rReverse transcription kit (Applied Biosystems), according to the manufacturer’s protocol. *miR-92a* and *miR-23a* in plasma were detected using Taqman miRNA assays (Applied Biosystems) on a 7500 HT real-time polymerase chain reaction machine (Applied Biosystems). Cel-*miR-39* was used as an endogenous control and the subsequent RNA isolation was then performed according to the manufacturers’ recommendation (final elution volume 50 μl). The addition of cel-*miR-39* allows controlling sample-to-sample variations of the RNA isolation efficiency; it was demonstrated previously that estimation of cel-*miR-39* is a suitable approach for normalization of plasma miRNAs in coronary heart disease [15].

### Statistical analysis

Data analysis was performed using the software of IBM SPSS Statistics version 18.0. Variables were evaluated using the Kolmogorov–Smirnov test or Shapiro–Wilks test to assess normality. Normally distributed data were expressed as the mean ± S.D. or as numbers. Comparison among groups was assessed by the chi-square test or ANOVA. Spearman’s correlation test was used to analyze correlations between EMPs and *miR-92a*. Each cardiac biomarker was examined; receiver operating characteristic (ROC) curves and optimal cut-off values were obtained. ROC curves were used for evaluation of diagnostic accuracy of EMPs, *miR-92a*, and cTnI. A value of *P*<0.05 was considered significant. Besides, the sensitivity and specificity value of the candidate biomarkers were determined.

## Results

### Characteristics of study subjects

In the present study, a total of 37 patients with AMI, 42 patients with SCAD, and 35 healthy people were recruited. As shown in Table 1, there were no significant differences in the following clinical characteristics between patients with AMI and patients with SCAD: age, gender, body mass index (BMI), hypertension, diabetes, history of smoking, total cholesterol (TC), low density lipoprotein cholesterol (LDL-C), high density lipoprotein cholesterol (HDL-C), and triglyceride (TG). Patient characteristics are detailed in Table 1.

**Table 1:** Clinical data of healthy subjects, SCAD, and AMI patients

	Controls	SCAD	AMI	*P*-value
	(*n*=35)	(*n*=37)	(*n*=42)	
Gender (% males)	57.1	59.5	66.7	0.083
Age (mean ± S.D.)	52.6 ± 5.1	53.7 ± 5.6	52.8 ± 5.6	0.125
BMI	23.1 ± 2.2	23.8 ± 2.9	24.2 ± 2.9	0.255
Smoker (%)	20.0	21.6	23.8	0.130
Diabetes mellitus (%)	5.7	18.9	19.0	0.314
TC (mmol/l)	4.4 ± 0.4	4.3 ± 0.6	4.4 ± 0.8	0.268
LDL-C (mmol/l)	2.4 ± 0.4	3.1 ± 0.8	3.5 ± 0.9	0.091
HDL-C(mmol/l)	1.4 ± 0.2	1.3 ± 0.3	1.2 ± 0.3	0.132
TG (mmol/l)	1.1 ± 0.2	1.5 ± 0.4	1.5 ± 0.5	0.178
ACE-I/ARB (%)	-	21.6	14.3	-
Calcium-channel blockers (%)	-	32.4	28.6	-
β-blockers (%)	-	64.8	69.0	-
Statin (%)	-	59.5	64.3	-

Values are described as mean ± S.D. or as the number of subjects. ACE-I/ARB: angiotensin-converting enzyme inhibitor/angiotensin II receptor blocker. Comparison was made between SCAD and AMI group and *P*-value was shown, a value of *P*<0.05 was considered with significant difference.

### Expression of MPs in plasma

**Figure 1:**
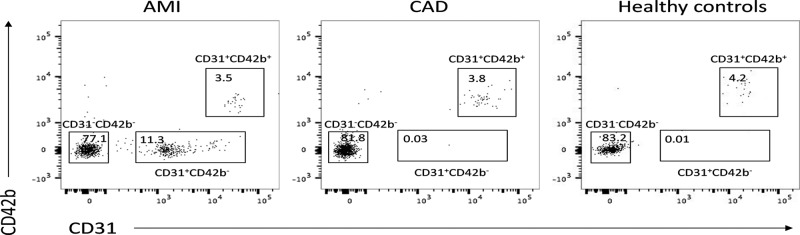
Representative flow cytometry analysis of MPs in plasma

As shown in Figure 1 and Table 2, we found the CD31+/CD42b− MPs were markedly higher in patients with AMI than patients with SCAD (*P*=0.0003) and controls. Besides, there was no significant difference between patients with SCAD and controls. However, CD31+/CD42b+ MPs and CD31−/CD42b− MPs showed no significant difference among the three groups.

**Table 2:** Characteristics of MPs in healthy subjects and patients with AMI or CAD

Characteristics	AMI	CAD	Healthy controls	*P*-value
CD31+/CD42b− MPs	12.5 ± 5.7	0.08 ± 0.07	0.04 ± 0.03	0.0003^*^
CD31+/CD42b+ MPs	3.4 ± 2.4	3.9 ± 1.8	3.8 ± 2.1	0.18
CD31−/CD42b− MPS	79 ± 8.9	82 ± 11.9	84 ± 10.6	0.37

Comparison was conducted between SCAD and AMI group and *P*-value was shown, * a value of *P*<0.05 was considered with significant difference.

### Expression of *miR-92a* and *miR-23a* in plasma

**Figure 2:**
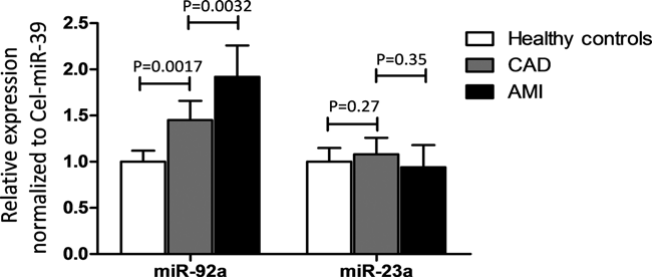
The relative expression of *miR-92a* and *miR-23a* in healthy subjects and patients with CAD or AMI Comparisons were made between CAD and healthy group, or CAD and AMI group. A value of *P*<0.05 was considered with significant difference.

Two coronary heart disease-related miRNAs, *miR-92a* and *miR-23a*, expressed differently among the three groups. As shown in Figure 2, the expression of *miR-92a* rather than *miR-23a* between patients with SCAD and controls, and between patients with AMI and SCAD both showed a significant difference.

### Correlations between circulating EMPs and *miR-92a* in AMI patients

**Figure 3:**
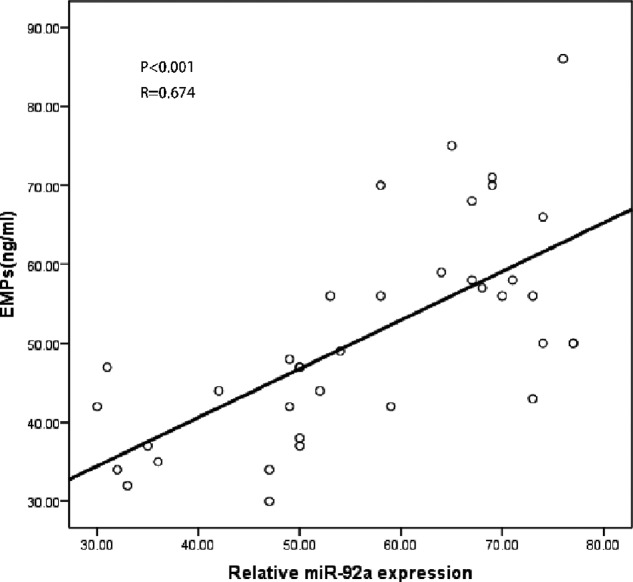
Correlation of the EMPs and *miR-92a* in AMI patients

To evaluate the possible link between EMPs and *miR-92a* in AMI patients, we analyzed whether there was a correlation between the high levels of expression of *miR-92a* and EMPs. It was revealed that the level of expression of *miR-92a* was significantly correlated with EMPs (see in Figure 3).

### Circulating EMPs and *miR-92a* expression as potential predictor of AMI

**Figure 4:**
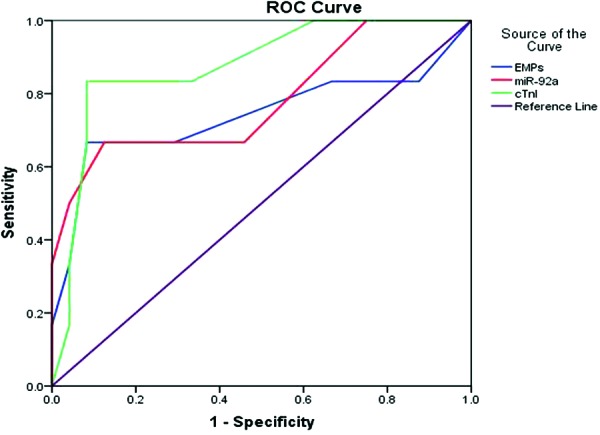
ROC curve analyses of EMPs and *miR-92a* in AMI patients

To further investigate the efficiency of EMPs and *miR-92a* as potential biomarkers of AMI, we performed ROC curve analysis between patients with AMI and patients with SCAD. Surprisingly, according to the outcome of ROC curve analysis, we found that the areas under the curve (AUC) of EMPs,* miR-92a*, and cTnI were 0.893, 0.888, and 0.912 respectively (see in Figure 4).

## Discussion

The high level of EMPs in plasma directly demonstrates a condition of endothelial dysfunction. Endothelial dysfunction has been associated with increased circulating EMPs [16], and high level of EMPs in plasma indicates endothelial activation [17]. In the present study, the level of EMPs was highly expressed in AMI rather than in SCAD, which indicated that the extent of endothelial cells activation was higher in AMI than in SCAD. In our study, for the first time we found that the expression of *miR-92a* in plasma was higher in AMI than SCAD, and in SCAD than controls. Since high level of *miR-92a* expression promotes endothelial activation and the development of atherosclerotic lesions, and *miR-92a* can serve as a valuable therapeutic target in angiogenesis and functional recovery of ischemic tissues in mice [18], we speculated that *miR-92a* might probably relate to myocardial damage. The mechanism under this hypothesis is unknown yet, but we postulated that endothelial cells-derived *miR-92a* might be transferred by EMPs from endothelial cells to myocardial cells and thus cause damage to the myocardial cells.

Furthermore, we explored the relationship between EMPs and *miR-92a* in AMI patients. It is known that EMPs have intricate functions in intercellular communication and compound exchange and can regulate target cells by carrying certain non-coding RNA and protein into them [19]. Interestingly, we showed a significantly positive correlation between EMPs and *miR-92a*, which implied that EMPs might regulate endothelial dysfunction and other types of cells through *miR-92a* in AMI.

Last, we examined the diagnostic value of EMPs and *miR-92a* in discriminating patients with AMI from patients with SCAD. Our results showed that the AUC of circulating EMPs, *miR-92a*, and cTnI were 8.925, 8.883, and 9.123 respectively. This evidence demonstrated that the high levels of expression of *miR-92a* and EMPs could be potential biomarkers to distinguish patients with AMI from patients with SCAD. However, since the sample size in the present study was relatively small, larger clinical studies are required to thoroughly establish the case.

## Conclusion

The present study indicates that the high level of plasma EMPs and* miR-92a* could reflect the extent of endothelial cells activation and more importantly, they could serve as novel biomarkers to diagnose patients with AMI from stable coronary heart disease.

## Abbreviations

AMI: acute myocardial infarction
CD31: Platelet endothelial cell adhesion molecule-1
cel-miR-39: Caenorhabditis elegans microRNA 39
CD42: cluster of differentiation 42
MI: myocardial infarction
AUC: areas under the curve
BMI: body mass index
CAD: coronary artery disease
cTnI: cardiac troponin I
EMP: endothelial microparticle
HDL-C: high density lipoprotein cholesterol
LDL-C: low density lipoprotein cholesterol
MP: microparticle
PFP: platelet-poor plasma
ROC: receiver operating characteristic
SCAD: stable coronary artery disease
TC: total cholesterol
TG: triglyceride
